# Impact of Age at Administration, Lysosomal Storage, and Transgene Regulatory Elements on AAV2/8-Mediated Rat Liver Transduction

**DOI:** 10.1371/journal.pone.0033286

**Published:** 2012-03-13

**Authors:** Gabriella Cotugno, Patrizia Annunziata, Maria Vittoria Barone, Marianthi Karali, Sandro Banfi, Alberto Auricchio

**Affiliations:** 1 Telethon Institute of Genetics and Medicine, Naples, Italy; 2 Medical Genetics, Dept. of Pediatrics, “Federico II” University, Naples, Italy; 3 Department of Pediatrics and European Laboratory for the Investigation of Food-Induced Diseases, “Federico II” University, Naples, Italy; 4 Medical Genetics, Dept. of General Pathology, Second University of Naples, Naples, Italy; University of Chicago, United States of America

## Abstract

Liver-directed gene transfer is being investigated for the treatment of systemic or liver-specific diseases. Recombinant vectors based on adeno-associated virus serotype 8 (AAV2/8) efficiently transduce liver cells allowing long term transgene expression after a single administration in animal models and in patients.

We evaluated the impact on AAV2/8-mediated rat liver transduction of the following variables: i) age at vector administration, ii) presence of lysosomal storage in liver cells, and iii) regulatory elements included in the transgene expression cassette. We found that systemic administration of AAV2/8 to newborn rats results in vector genome dilution and reduced transduction efficacy when compared to adult injected animals, presumably due to hepatocyte proliferation. Accumulation of glycosaminoglycans in lysosomes does not impact on levels and distribution of AAV2/8-mediated liver transduction. Transgene expression occurs in hepatocytes but not in Kupffer or liver endothelial cells when the liver-specific thyroxine-binding-globulin promoter is used. However, extra-hepatic transduction is observed in the spleen and kidney of animals injected at birth. The use of target sequences for the hematopoietic-specific microRNA miR142-3p does not improve liver transduction efficacy neither reduce immune responses to the lysosomal enzyme arylsulfatase B. The inclusion of a variant of the Woodchuck hepatitis virus post-transcriptional regulatory element (WPRE-m) decreases AAV2/8-mediated liver transduction levels.

As AAV2/8-mediated liver gene transfer is entering in the clinical arena, these data will provide relevant information to the design of efficient AAV2/8-based therapeutic strategies.

## Introduction

The liver is the largest organ in the body performing essential functions in metabolism, detoxification, and production of plasma proteins [1]. The liver consists of several cell types classified into hepatocytes (liver parenchymal cells) that constitute about 80% of liver cells [2] and non-parenchymal cells represented by endothelial cells, Kupffer cells (resident liver macrophages), fat-storing cells (stellate cells or Ito cells), and pit cells (natural killer cells). Kupffer and endothelial cells form the hepatic reticulo-endothelial system [3] and constitute the majority of liver non-parenchymal cell types [2]. Given its central role in maintaining homeostasis and regulating metabolism, and the high accessibility of hepatocytes via the bloodstream through a fenestrated epithelium, the liver has been widely utilized for the expression of therapeutic transgenes via somatic gene transfer [4], [5]. This approach has been used to treat several inherited and acquired disease models, either affecting the liver itself or requiring systemic delivery of a therapeutic protein, such as hemophilia, lysosomal storage disorders, and others [4], [5].

Vectors based on the adeno-associated virus serotype 8 (AAV2/8) hold promise for *in vivo* liver directed gene transfer [4], [5]. Systemic administration of AAV2/8 results in long-term, robust transgene expression with minimal toxicity and immune responses in several animal models [4], [5]. In addition, preliminary results from an ongoing clinical trial using AAV2/8 in hemophilia B patients suggest that systemic AAV2/8 administration is safe and efficient in humans [6], [7]. The AAV vector genome persists mainly as an episome in transduced hepatocytes with only a small percentage being integrated in the host cell genome [5], [8], [9]. While this is associated with a low risk of insertional mutagenesis, it may represent a limitation for the transduction of actively replicating cells as in the case of newborn tissues [4].

Moreover, liver-directed gene delivery has been reported to induce transgene product-specific immune tolerance [1]. This is related to the unique cellular environment of the liver, which is physiologically involved in the appropriate recognition of self versus non-self molecules and pathogens [10], [11]. Reduced or absent activation of the immune system after vector administration represents an advantage for the induction of transgene-specific tolerance [1]. The low immunogenicity of AAV vectors, partly due to their inefficiency in infecting dendritic cells (DC) or macrophages, favours tolerance induction [1]. Despite this, examples of transgene-directed immune responses following liver gene delivery have been reported [4], [5], [12]–[15]. The tolerance obtained after liver gene delivery requires optimization of gene transfer strategies to eliminate transgene expression in antigen presenting cells (APC) while restricting high levels of therapeutic expression to hepatocytes [1].

The choice of regulatory elements to be included in the transgene expression cassette has a direct impact on the levels and cellular restriction of gene expression. This is crucial to improve both therapeutic efficacy and safety, and to avoid the development of transgene-directed immune responses in liver gene transfer protocols. Hepatocyte-specific promoters, such as the thyroxine-binding-globulin promoter (also called liver-specific, TBG or LSP [16]–[25]), are often used to obtain liver-restricted transgene expression. In addition, the inclusion of microRNA (miRNA) target sequences in the vector expression cassette can help to eliminate off-target transgene expression from transduced cells that express the corresponding miRNA [26]. Moreover transgene expression levels can be improved by the inclusion, in the transgene expression cassette, of post-trascriptional regulatory elements such as the Woodchuck hepatitis virus (WHV) post-transcriptional regulatory element (WPRE, [27]), able to increase transcript levels and/or stability.

Equally important is the timing of vector administration, as well as the pathological conditions, that account for alterations of the microenvironment and cell function of the liver, which could affect the efficacy and safety of liver gene transfer [4], [28].

Here we investigated the impact of the following factors on the efficacy of AAV2/8-mediated liver gene transfer in rats, commonly used as animal models for the development of gene therapy strategies: i) age at vector administration, ii) presence of lysosomal storage in liver cells, and iii) regulatory elements included in the transgene expression cassette.

## Results

### AAV2/8 systemic delivery in newborn rats is associated with vector dilution and low transduction levels

**Figure 1:**
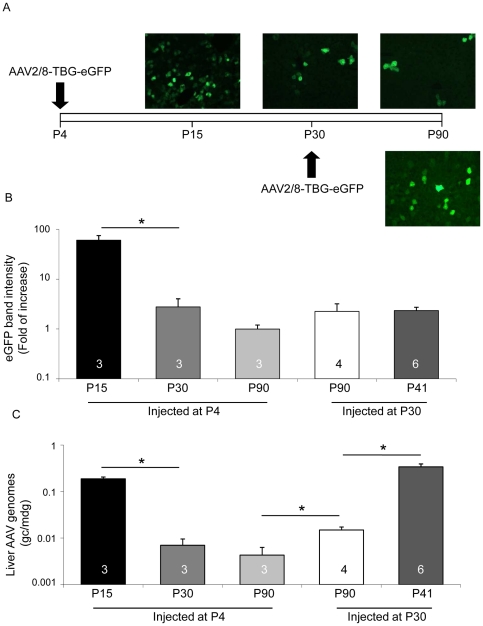
AAV2/8 administration to newborn rats is associated with vector genome dilution. A) Wild-type rats were injected at either postnatal day 4 (P4, upper pictures), or at P30 (lower picture) with 4×10e13 gc/kg of AAV2/8-TBG-eGFP. Animals injected at P4 were sacrificed at P15, P30 or P90, while those injected at P30 were analysed at P90. EGFP expression was confirmed under a fluorescence microscope on sections from transduced livers. Representative pictures from animals in each group are shown. Magnification: 20×. B) The eGFP expression levels were analyzed by Western blot of liver lysates from AAV-injected rats. The intensity of eGFP band was quantified, normalized on the corresponding tubulin band and expressed as fold of increase in each animal group compared to rats injected at P4 and sacrificed at P90. Results are reported as mean ± SE. The number of rats analyzed in each group is reported in each bar. *: p<0.05. The fold of increase of eGFP expression in livers from rats injected at P30 (white bar) compared to rats injected at P4 (light grey bar) and collected at P90 is 2.25±0.95. C) AAV vector genome copies per molecule of diploid genome (gc/mdg) were measured by Real-time PCR in Hirt DNA extracted from injected rats. Results are reported as mean ± SE. The number of rats analyzed in each group and statistical significance is reported as before.

To assess the impact of rat hepatocyte proliferation on AAV2/8-mediated liver transduction, we injected rats systemically with AAV2/8 vectors encoding the enhanced Green Fluorescent Protein (eGFP) transgene under the control of the liver-specific TBG promoter (AAV2/8-TBG-eGFP) either at postnatal day (P) 4 or at P30, assuming that the rate of hepatocyte proliferation is reduced at P30 [29]. Livers from animals administered with AAV were collected at P90 and levels of transgene expression were assessed by both analysis of eGFP-positive cells on cryo-sections (Fig. 1A and Fig. S1A) and anti-eGFP Western blot (Fig. 1B, Fig. S1B and Fig. S2A). We observed a higher number of eGFP-expressing cells (Fig. 1A, right panels and Fig. S1A) and a stronger (but not significantly different) intensity of eGFP bands (Fig. 1B, P90 white and grey bars, Fig. S1B and Fig. S2A) in livers from animals injected at P30 compared to those injected at P4. Concordantly, the number of AAV genome copies/molecule of diploid genome (gc/mdg) was significantly higher in livers from animals injected at P30 than in those injected at P4 (Fig. 1C, P90 white and grey bars).

To confirm that the reduced amount of vector genomes and the lower transgene expression levels observed in livers of rats injected at P4, as opposed to those injected at P30, were due to the proliferation-related dilution of episomal AAV vector genomes, we injected rats at P4 with AAV2/8-TBG-eGFP and collected livers at P15, P30 and P90. We observed the highest number of eGFP-positive cells (Fig. 1A), highest eGFP expression levels (Fig. 1B and Fig. S2B) and vector gc/mdg (Fig. 1C) in livers collected at P15. These were reduced in animals sacrificed at later time points with the strongest reduction observed between P15 and P30. In addition, the eGFP-positive cells in livers collected at P90 appeared in clusters when rats were injected at P4 but not at P30 (data not shown), possibly representing clones from a single cell containing integrated vector genomes, as previously described in mice injected at birth with AAV2/8 [30]. Thus, as previously reported [9], [30], [31], hepatocyte proliferation results in dilution of AAV vector genomes in transduced cells and reduced efficacy of long-term liver transduction in newborn rats.

To test if AAV vector genome dilution occurs in liver of adult animals, we injected rats with AAV2/8-TBG-eGFP at P30 and collected their livers at P41 and at P90. Western blot analysis showed in some but not all the livers collected at P41 slightly higher levels of eGFP expression than at P90 (Fig. S2C). However the quantification of the corresponding eGFP band intensity did not evidence statistically significant differences (Fig. 1B, white and dark grey bars). Thus, although we can't exclude that in some animals vector dilution occurs in liver of rats injected at P30, overall this was not significant. Interestingly, the amount of liver vector genomes at P41 is significantly higher than at P90 (Fig. 1C, white and dark grey bars). Since these differences are not reflected by the transduction levels, we hypothesize that some of the AAV genomes in liver at P41 are single-stranded and thus transcriptionally inactive, and are then lost at P90.

Overall, liver transgene expression levels seem higher 11 days after vector injection at P4 (Fig. S2B, P15) than at P30 (Fig. S2C, P41) suggesting that AAV2/8-mediated liver transduction is more efficient in newborn than in adult rat liver.

### Lysosomal storage does not reduce the efficacy of AAV2/8-mediated hepatocyte transduction

**Figure 2:**
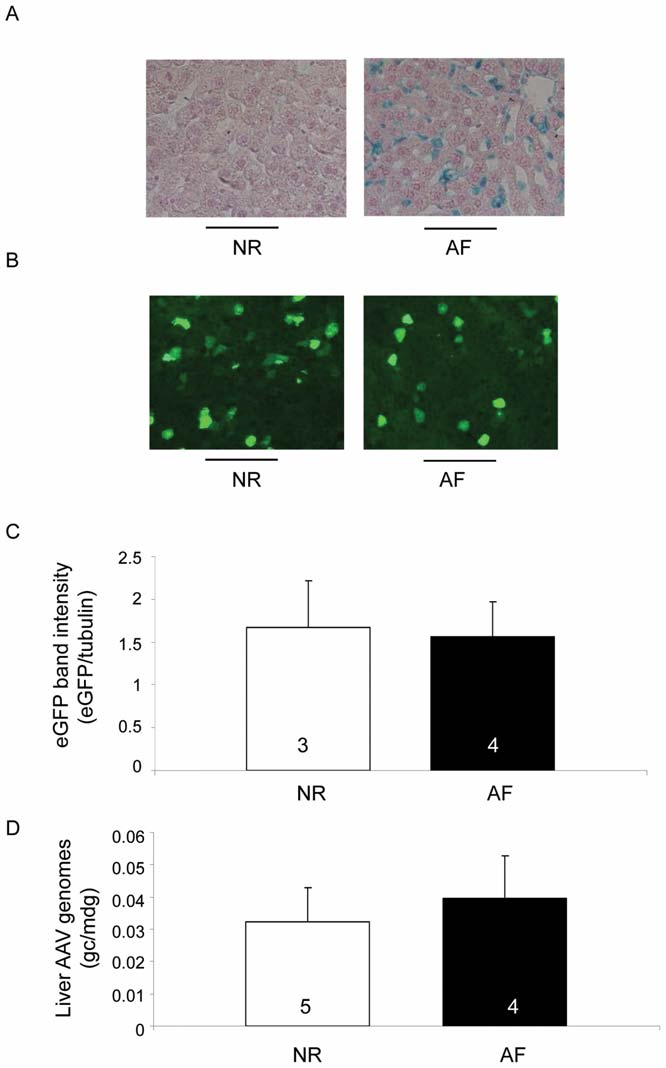
Lysosomal storage does not impact on AAV2/8-mediated liver transduction. A) Alcyan blue staining performed on liver sections from wild-type (NR) and MPS VI (AF) rats injected with AAV2/8-TBG-eGFP shows GAG storage (blue staining) in AF but not NR rats. Representative pictures from animals in each group are shown. Magnification: 20×. B) NR and AF rats were injected at P30 with 4×10e13 gc/kg of AAV2/8-TBG-eGFP vectors. Animals were sacrificed at P90 and eGFP expression levels were assessed under a fluorescence microscope on sections from transduced livers. Representative pictures from animals in each group are shown. Magnification: 20×. C) eGFP expression levels were assessed by Western blot analysis of liver lysates from the same animals as in panel B. The intensity of eGFP bands was measured and normalized to that of the corresponding tubulin band. Results are reported as mean ± SE. The number of animals analyzed in each group is reported in each bar. D) AAV vector genome copies/molecule of diploid genome (gc/mdg) were measured by Real-time PCR in livers of rats injected with AAV2/8. Results are reported as mean ± SE. The number of animals analyzed is reported as before.

Lysosomal storage disorders (LSD) are excellent candidate diseases for gene therapy because: i) lysosomal enzymes are secreted by producing cells and uptaken by deficient cells [32], ii) relatively low levels (5–10% of normal) of lysosomal enzymes are expected to significantly reduce morbidity [33], and iii) enzyme replacement therapy that exists for several lysosomal enzymes requires costly, life-lasting infusions that do not completely resolve LSD. We have recently described the efficacy of AAV2/8-mediated liver gene transfer in animal models of mucopolysaccharidosis VI (MPS VI), an LSD characterized by glycosaminoglycan (GAG) storage in various tissues including liver (Fig. 2A) due to arylsulfatase B (ARSB) deficiency [14], [15], [34].

Here we set up to test if GAG storage has an impact on AAV2/8-mediated liver transduction using a rat model of MPS VI [14]. Normal (NR) and MPS VI affected (AF) rats were injected at P30 with AAV2/8-TBG-eGFP vectors. Livers were collected at P90 and the efficiency of AAV2/8-mediated transduction was assessed. We observed a similar number of eGFP-positive liver cells (Fig. 2B) and a comparable intensity of eGFP bands by Western blot on liver lysates from NR and AF rats injected with AAV2/8-TBG-eGFP (Fig. 2C and Fig. S2D). In addition, we did not detect any significant difference in the amount of liver AAV vector genomes between the two animal groups (Fig. 2D). This suggests that lysosomal storage does not significantly impact on AAV2/8-mediated liver transduction in MPS VI rats.

### Impact of transgene regulatory elements on AAV-mediated liver gene transfer

**Figure 3:**
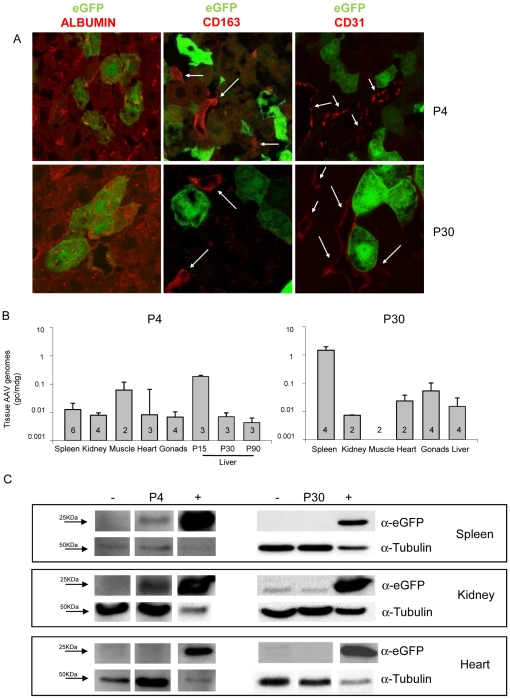
TBG-driven transgene expression in tissues of rats injected with AAV2/8. A) Hepatocyte-restricted eGFP expression in the liver of rats injected with AAV2/8-TBG-eGFP. Wild-type rats were injected at P4 (upper pictures), or at P30 (lower pictures) with 4×10e13 gc/kg of AAV2/8-TBG-eGFP vectors. Animals were sacrificed at P15 (those injected at P4) or at P90 (those injected at P30). The identity of eGFP expressing cells in the liver from injected rats was confirmed by confocal microscopy analysis on sections after immuno-fluorescence with anti-albumin (left panels), anti-CD163 (middle panels, white arrows) or anti CD-31 (right panels, white arrows) antibodies. Representative pictures from rats in each group are shown. Magnification: 63×. B, C) Analysis of vector bio-distribution and eGFP expression in tissues from rats injected with AAV2/8-TBG-eGFP. B) AAV vector genome copies/molecule of diploid genome (gc/mdg) were measured in tissues (reported under each bar) from rats injected at P4 (left, for spleen, kidney, muscle, heart and gonads data are pooled from tissues harvested at P15, P30 and P90) or at P30 (right, all tissues harvested at P90) by Real-time PCR. Results are reported as mean ± SE. The number of animals analyzed in each group is reported in the corresponding bar. C) eGFP expression was assessed by Western blot analysis on the spleen (70 µg of proteins) , kidney (70 µg of proteins) and heart (150 µg of proteins). Representative animals from each group are shown. −: tissues from uninjected rats; +: liver lysate from a rat injected at P4 with AAV2/8-TBG-eGFP and collected at P15 used as eGFP-positive control (10 ug of proteins).

The TBG or Liver-Specific (LSP) synthetic promoter is often used in the context of liver gene transfer [14]–[25], [34]. To assess whether TBG drives transgene expression specifically to hepatocytes after systemic administration of AAV2/8, we injected rats at P4 or at P30 with 4×10e13 gc/kg of AAV2/8-TBG-eGFP. Tissues from injected and uninjected animals were collected at the same time points reported in the previous section and vector biodistribution as well as eGFP expression levels were analyzed in the liver, spleen, kidney, muscle, heart and gonads. Liver cryo-sections were stained with anti-albumin to identify hepatocytes (Fig. 3A, left panels, red signal), anti-CD163 to mark Kupffer cells (Fig. 3A, middle panels, red signal) or anti-CD-31 to label endothelial cells (Fig. 3A, right panels, red signal) and analyzed by confocal microscopy to co-localize eGFP with one of the three markers (Fig. 3A). eGFP co-localized with albumin but not with CD163 or CD-31, independently of the age at vector administration (Fig. 3A) suggesting that AAV2/8-TBG vector administration results in hepatocyte-specific transduction in the rat liver. Similar results were obtained in MPS VI rats injected at P30 with AAV2/8-TBG-eGFP vectors (data not shown), suggesting that lysosomal storage does not alter the pattern of AAV2/8-TBG-mediated liver transduction. The low levels of endothelial cell transduction mediated by AAV2/8-TBG were additionally observed in human cell lines. Human hepatoma (HepG2) and human umbilical vein endothelial (HUVEC) cells were infected with 1×10e5 (data not shown) or 5×10e5 gc/cell of AAV2/8-TBG-eGFP vectors or vectors encoding eGFP under the control of the ubiquitous chicken beta-actin promoter (AAV2/8-CBA-eGFP). The percentage of eGFP-expressing cells was determined by FACS analysis. While both HepG2 and HUVEC cells were transduced efficiently when using AAV2/8-CBA-eGFP, HepG2 but not HUVEC cells were transduced by AAV2/8-TBG-eGFP (Fig. S3).

AAV vector genomes were detected by Real-time PCR in the spleen, kidney, muscle, heart and gonads of rats injected either at P4 (collected at P15, P30 and P90) or at P30 (collected at P90) (Fig. 3B). Western blot analysis with anti-eGFP antibodies of lysates from the same tissues did not show detectable eGFP in the muscle, heart and gonads of treated rats, independently of the age at vector administration (Fig. 3C, lower panels and data not shown). However, low levels of eGFP protein (Fig. 3C, left panels) and transcript (Fig. S4) were detected in the spleen and kidney from rats injected at P4, suggesting that TBG-driven expression is not restricted to hepatocytes when AAV2/8 is administered systemically to newborn rats. Interestingly, while TBG-driven eGFP expression was detected in the spleen until P90 after vector administration (the last time point of the study), ectopic eGFP expression in the kidney was undetectable after P15 (data not shown). Inclusion of target sequences for the hematopoietic lineage-specific miRNA miR142-3p (miR142-T) in the 3′UTR of the transgene expression cassette has been reported to de-target transgene expression from hematopoietic cells in the spleen [35]. To test if this strategy can be exploited to inhibit ectopic transgene expression in the spleen of rats injected at P4 with AAV2/8-TBG vectors, we generated AAV2/8-TBG-eGFP vectors carrying four copies of the miR142-T (miR142-Tx4) immediately downstream of the eGFP coding sequence (see Materials and Methods). Wild-type rats were injected at P4 with 4×10e13 gc/kg of either AAV2/8-TBG-eGFP or AAV2/8-TBG-eGFP-miR142-Tx4. Eleven days after vector delivery, Western blot analysis of eGFP expression showed transgene expression in liver but not spleen of rats receiving AAV2/8-TBG-eGFP-miR142-Tx4 (Fig. S2F) confirming that the inclusion of miR142-T prevents transgene expression in this ectopic site. We have also observed that liver eGFP expression was lower in rats receiving vectors containing miR142-T compared to those injected with AAV2/8-TBG-eGFP (Fig. S2F and S2B respectively). We speculate that the addition of the miR142-T sequence to the eGFP transcript may reduce its stability.

Inclusion of the WPRE sequence in the 3′ UTR of the transgene expression cassette has been reported to increase expression levels in the context of various viral vectors and cell types [36]–[51]. However, concerns have been raised regarding the inclusion of WPRE in viral vectors because its sequence overlaps with that of the WHV X protein (WHX), a transcriptional activator implicated in the development of liver tumors [52], [53]. To avoid expression of WHX-derived polypeptides, a version of WPRE mutated in both the WHX promoter and the translation start sequence has been generated which has an efficacy in transgene expression enhancement similar to that of the wild-type WPRE sequence [51], [54]. We tested the impact of this mutant WPRE variant (WPRE-m, bp 1094–1636, GenBank J04514 and [51]), on transgene expression levels both in the human HepG2 hepatoma cell line and in the rat liver.

**Figure 4:**
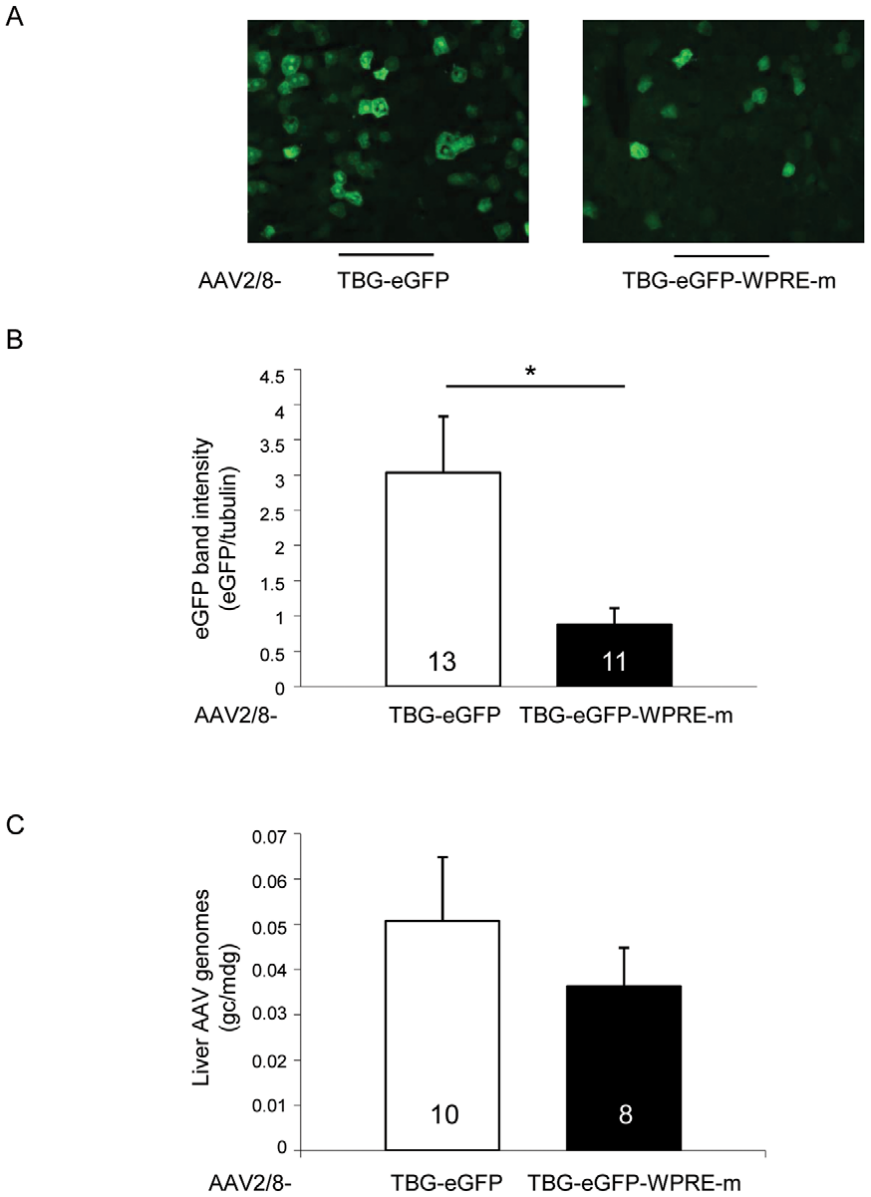
Inclusion of the WPRE-m variant is associated with decreased hepatocyte transduction levels in rats. A) Rats were injected at postnatal day (P)30 with 4×10e13 gc/kg of AAV2/8-TBG-eGFP or AAV2/8-TBG-eGFP-WPRE-m vectors. Animals were sacrificed at P90 and eGFP-positive cells were visualized on liver sections under a fluorescence microscope. Representative pictures from animals in each group are shown. Magnification 20×. B) eGFP expression levels were assessed by Western blot analysis on liver lysates from the same animals as in panel A. The intensity of eGFP bands was measured and normalized on that of the corresponding tubulin band. Results are reported as mean ± SE of three independent experiments. The number of animals analyzed in each group is reported inside each bar. *: p<0.05. C) AAV vector genome copies/molecule of diploid genome (gc/mdg) were measured by Real-time PCR in livers of rats injected with AAV. Results are reported as mean ± SE of three independent experiments. The number of animals analyzed in each group is reported inside each corresponding bar. The p-value between the TBG-eGFP and TBG-eGFP-WPRE-m groups is 0.44.

HepG2 cells were transfected with expression plasmids encoding eGFP or feline ARSB (fARSB) under the control of the TBG promoter, containing or not the WPRE-m element (pAAV-TBG-eGFP, pAAV-TBG-eGFP-WPRE-m, pAAV-TBG-fARSB, pAAV-TBG-fARSB-WPRE-m). Similar levels of eGFP [eGFP (OD): 274±0.4 for pAAV-TBG-eGFP and 301±18 for pAAV-TBG-eGFP-WPRE-m transfected cells] or normalized ARSB activity [ARSB(nmol/mg/h)/eGFP(OD): 0.36±0.04 for pAAV-TBG-fARSB and 0.37±0.05 for pAAV-TBG-fARSB-WPRE-m transfected cells] were measured independently of the WPRE-m inclusion. We then injected rats at P30 with AAV2/8-TBG-eGFP vectors including or not the WPRE-m variant and assessed transgene expression levels in livers collected at P90. We analyzed both the presence of eGFP positive cells on cryo-sections by fluorescence microscopy and eGFP levels of expression in liver lysates by Western blot with anti-eGFP antibodies (Fig. 4A and B, Fig. S2E and Fig. S5A). Despite the presence of similar amounts of AAV vector genomes (Fig. 4C and Fig S5B), the levels of eGFP expression appeared lower in livers receiving the vectors with than without WPRE-m (Fig. 4A and B, Fig. S2E and Fig. S5A). To exclude that the differences observed in eGFP expression levels were due to the quality of viral vector preparations, three independent experiments using 3 different preps of vector for each construct were performed and showed similar results (Fig. 4A and B, Fig. S2E and Fig. S5A).

**Figure 5:**
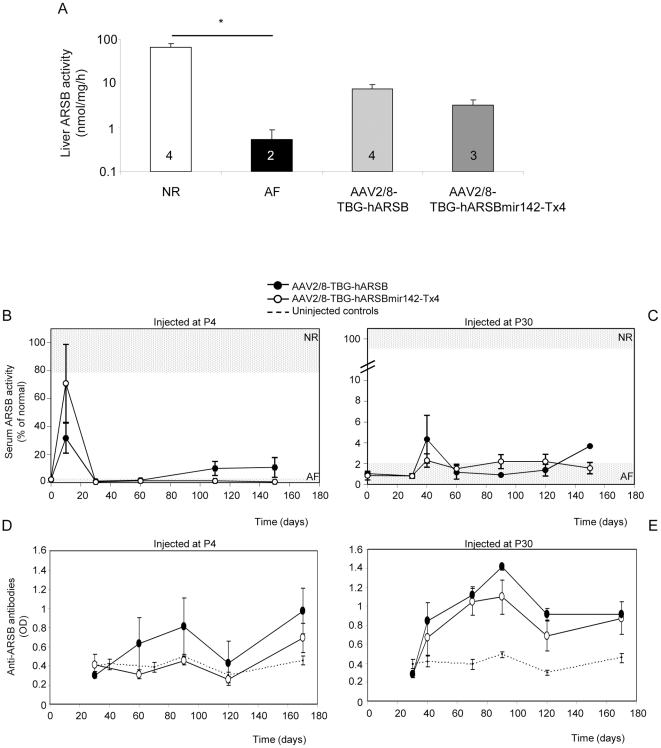
Inclusion of target sequences for miR142-3p in the AAV vector genome does not reduce immune responses to ARSB in MPS VI rats injected with AAV2/8-TBG-hARSB. MPS VI rats were injected either at P4 or at P30 with AAV2/8-TBG-hARSB or AAV2/8-TBG-hARSB-miR142-Tx4 vectors. Animals were followed up for 6 months after vector delivery when they were sacrificed for analysis of tissue ARSB expression. A) Liver ARSB activity was measured at the time of sacrifice in rats injected at P4 with AAV2/8-TBG-hARSB or AAV2/8-TBG-hARSB-miR142-Tx4. The number of animals analyzed in each group is reported in each bar. NR: control rats AF: uninjected MPS VI rats. * = p<0.05. The p-value between the TBG-hARSB and TBG-hARSB-miR142-Tx4 groups is 0.12. B, C) Serum ARSB activity was measured each month in NR and AF rats (light grey horizontal grids) and in rats injected with either AAV2/8-TBG-hARSB or AAV2/8-TBG-hARSB-miR142-Tx4 vectors at P4 (B) or at P30 (C). Results are reported as mean ± SE for each group. D, E) Serum anti-ARSB antibodies were measured by ELISA each month in AF rats injected with either AAV2/8-TBG-hARSB or AAV2/8-TBG-hARSB-miR142-Tx4 vectors at P4 (D) or at P30 (E). As negative control anti-ARSB antibodies were measured in sera from NR and AF rats that did not receive AAV vectors (uninjected controls). Results are reported as mean ± SE for each group.

Neutralizing humoral immune responses to a secreted transgene product has often limited the efficacy of liver-directed gene transfer [14], [15], [26], [55], [56]. In some instances, this has been solved using liver-specific promoters [1] presumably by avoiding ectopic transgene expression in APC. In addition, age at vector administration has been shown to impact on levels of immune reponses to the transgene product [57]. Indeed, as we have previously reported [15], serum ARSB activity measured over time in null MPS VI rats receiving AAV2/8-TBG vectors encoding human ARSB (hARSB) at P4 were higher than those measured in rats injected with the same vectors at P30 (Fig. 5B and 5C). This correlates with the lower levels of anti-ARSB antibodies developed in rats injected as newborn compared to those injected at P30 (Fig. 5D and E and [15]). The extent of the humoral immune response may thus explain the lower circulating ARSB activity detected in animals treated at P30 compared to those injected at P4.

The development of neutralizing antibodies to ARSB in null MPS VI rats was not completely prevented neither by using a liver-specific promoter nor by administering vectors at P4 (Fig. 5D and E and [15]). Only when immuno-suppressant drugs were co-administered with AAV vectors, the production of systemic antibodies to ARSB was inhibited and higher levels of circulating ARSB were obtained [15]. More recently, inclusion of miR142-T sequences in the transgene expression cassette has been used to avoid off-target transgene expression in APC and prevent transgene-directed immune responses in the context of liver-directed gene transfer [35]. To avoid the use of immuno-suppressant drugs, we tested whether inhibition of ectopic transgene expression through the inclusion of miR142-Tx4 in our vectors could prevent the development of transgene-directed immune responses to ARSB in MPS VI rats. MPS VI rats were injected either at P4 or at P30 with 4×10e13 gc/kg of either AAV2/8-TBG-hARSB or AAV2/8-TBG-hARSB-miR142-Tx4. Animals were followed-up for 6 months after vector delivery and were then sacrificed for analysis of ARSB expression in liver. The levels of liver ARSB activity were similarly increased in rats receiving AAV2/8 vectors at P4 compared to AF controls, independently of the inclusion of the miR142-Tx4 element (Fig. 5A). This is different from what we observed when the miR142-Tx4 sequence was included in the eGFP encoding vectors, resulting in reduced levels of liver transgene expression (Fig. S2). However this is not surprising since the miR142-Tx4 sequence may negatively impact on the stability of transcripts deriving from the eGFP but not from the ARSB expression cassettes used in this study. Monthly serum ARSB activity was very low in rats treated as newborns, independently of the presence of miR142-T sites (Fig. 5B). Indeed, serum anti-ARSB antibodies could be measured in rats receiving the miR142-3p-regulated vector, even though at slightly lower levels than those injected with AAV2/8-TBG-hARSB vectors (Fig. 5D). Similar data were obtained in rats injected at P30 (Fig. 5C and E), suggesting that the inclusion of binding sites for miR142-3p in AAV2/8-TBG-hARSB vectors does not avert the development of anti-ARSB humoral immune responses.

## Discussion

Given the growing interest in AAV2/8-mediated liver gene transfer, here we evaluated in rats the impact of variables commonly affecting the efficacy and specificity of hepatocyte transduction.

For several severe and progressive diseases, early treatment is required to achieve therapeutic efficacy. We previously reported peak-and-drop kinetics of transgene expression after AAV2/8-mediated liver neonatal gene transfer in MPS VI rats and cats [15], [34]. The results described here suggest that AAV vector dilution occurs in the first weeks after gene delivery in newborn rats and is associated with strong reduction of transgene expression levels, as previously reported [4], [30], [31]. This is expected given the high rate of hepatocyte proliferation in the neonatal period [29] and the episomal status of recombinant vector genomes in cells transduced with AAV [8], [9]. However additional factors, i.e. cytotoxic immune responses to AAV-transduced cells, should not be completely ruled out. Differently from newborn liver, murine adult hepatocytes are replaced very slowly, approximately once every 180–400 days [58]. Indeed, delivery to adult murine hepatocytes allows to obtain robust long-term transgene expression [4], [9]. Consistent with this, we observed higher transgene expression levels in the livers of animals injected at P30 than those treated at P4. Since the rat liver mass increases for at least 100 days after birth [29], we tested if AAV vector dilution could also occur in the liver of rats injected at P30. Although we observed a reduction in the amount of rat liver vector genomes over time after AAV delivery at P30, we could not detect significant differences in transgene expression levels. A possible explanation for this, could be that single-stranded, transcriptionally inactive AAV genomes are lost between P41 and P90.

Liver gene transfer has been used to create a depot organ for long-term release of lysosomal enzymes as a potential therapeutic strategy for LSD [4], [5]. Lysosomal storage causes tissue inflammation, alteration of cellular degradation pathways such as autophagy and increased rate of apoptosis [59]–[61]. We show that this does not impact either on the levels or on the pattern of cell transduction within the liver parenchyma of AAV2/8 in MPS VI rats up to day 90, the last time point of the analysis. However, we can not exclude that loss of AAV genomes may occur later in the MPS VI rat liver as result of the presence of activated macrophages and increased apoptosis as we have previously reported [14], [61].

Regulation of gene expression in space and levels is required for safe and efficient gene therapy. Liver-specific promoters are often used to restrict viral vector-mediated expression to hepatocytes. As aberrant transgene expression has been reported with both synthetic and native hepatocyte-specific promoters [35], [62], we tested if this is also the case with the widely used TBG promoter. Although we found that AAV2/8-TBG-mediated transduction is restricted to hepatocytes in both wild-type and MPS VI liver, we observed detectable eGFP expression in the spleen and kidney of wild-type rats injected systemically with AAV2/8-TBG-eGFP at P4, suggesting that the TBG promoter is not hepatocyte-specific, at least in newborn rats. This partially agrees with data reported from Bell et al. and Wang et al., showing that low levels of eGFP mRNA are detected in the spleen of dogs [25] and non-human primates [22] injected sistemically with AAV2/8-TBG-eGFP. However, we can not exclude that the eGFP expression we observed in non-hepatic rat tissues may derive from the promoter activity associated with the AAV inverted terminal repeats (ITR) [63].

Interestingly, AAV vector genomes were higher in the spleen from rats injected at P30 compared to those injected at P4; thus, proliferation-related dilution of AAV vector genomes may occur in newborn spleen as observed for the liver. Alternatively, efficiency of adult and newborn spleen cells transduction after systemic AAV2/8 administration might be different. In addition, it is possible that different cell types are transduced in newborn and adult spleen after systemic AAV2/8-injection, and this could explain the absence of detectable eGFP expression in the spleen from rats injected at P30, despite the high number of gc/mdg. Off-target transgene expression in splenic APC cells after systemic injection of lentiviral vectors has been described to induce transgene-directed immune responses in the context of liver-directed gene transfer [35]. This was avoided by the inclusion of target sites for miR142-3p in the transgene expression cassette. Even though AAV vectors are considered overall inefficient at APC transduction [1], [64], transgene expression in DC or macrophages has been reported after AAV delivery [65], [66]. We hypothesized that this may contribute to the anti-hARSB immune responses we observed in MPS VI rats after liver transduction with AAV2/8-TBG-hARSB [14], [15] and that this could be prevented by the inclusion of the miR142-Tx4 element in the AAV2/8-TBG-hARSB expression cassette. However, the low ARSB serum levels and the high anti-ARSB antibody titers we found despite the use of the miR142-3p target sequence argue against this. This is not surprising since ARSB, like other lysosomal enzymes, is secreted from producing cells and can be efficiently uptaken via the mannose-6-phosphate receptor pathway from non-transduced cells [32], possibly including APC, thus resulting in immune system activation independently of APC transduction. In addition, the inflammatory process, which is associated with storage in MPS VI liver [14], [61] may favour the development of immune responses against the transgene [28].

The WPRE element has been included in gene therapy vectors with the goal of increasing transduction levels. However, the impact of WPRE inclusion on transgene expression is variable and depends on the context of the promoter [43], [67]–[69], the transgene [70], the gene transfer vector used [49], [54] and target cell type [42], [68], [69], [71]–[73]. Notably, in some instances the use of WPRE has been reported to decrease transgene expression levels [69], [72]–[75]. Indeed, we found that the inclusion of a WPRE variant (WPRE-m) either did not increase AAV-mediated transduction levels *in vitro* or resulted in reduced transgene expression in rat liver. Others have described increased transgene expression levels in murine liver using adenoviral or AAV vectors including WPRE [42], [45], [46]. The following differences with our study may be responsible for this apparent discrepancy: i) the rodent specie used, as others have used mice and we used rats; ii) the promoter/vector context which are different from our study; iii) the WPRE sequence as the WPRE-m we used is partially deleted in the β sub-element [27]. Indeed, work from Shambach et al. [54] and Donello et al. [27] has shown that WPRE devoid of the entire β sub-element has reduced activity *in vitro* (30 [27] to 75% [54] of normal). Interestingly, in two independent studies, the use of WPRE sequences lacking the β subunit has been shown to have detrimental effect on transgene expression levels in some subsets of hematopoietic cells [72], [73]. Thus it is possible that the WPRE-m sequence selected in our study is responsible for the observed detrimental effect on AAV2/8-mediated rat liver transduction. Since the WPRE-m element selected in this study is shorter than the WPRE used in studies where increased transgene expression levels were observed following AAV transduction [37], [38], [45], [47] the conclusions on the effect of WPRE-m should not be extended to other WPRE sequences.

In conclusion we show that: i) systemic AAV2/8 administration to newborn rats is associated with vector dilution and low transduction levels; ii) the combination of AAV2/8 with the hepatocyte-specific promoter TBG prevents transgene expression in liver Kupffer and endothelial cells but not in newborn kidney and spleen; iii) the inclusion of target sequences for the miR142-3p in the expression cassette of the lysosomal enzyme ARSB does not prevent humoral immune responses to the transgene, and iv) the inclusion of WPRE-m in AAV2/8-TBG vectors is associated with decreased rat liver transduction levels.

These data will be useful when designing liver gene therapy strategies based on systemic administration of AAV2/8.

## Materials and Methods

### Ethics Statement

All studies on rats were conducted in strict accordance with the institutional guidelines for animal research and with the Guide for the Care and Use of Laboratory Animals of the National Institutes of Health. All animal treatments were reviewed and approved by the Ethics Committee for Animal Experimentation (CESA, “Carlo Bo” University, Urbino, Italy) on May 17, 2007. All procedures on rats were then approved by the Italian Ministry of Health (protocol number: 581/07; approval date Sept. 09, 2007).

All surgery was performed under anesthesia, and all efforts were made to minimize suffering.

### Plasmids and vector production

The TBG synthetic promoter used in this study contains contains a human thyroid hormone-binding globulin promoter sequence (GenBank ID: NT011651.17) and two copies of an α1-microglobulin/bikunin enhancer sequence (GenBank ID: NT008470.19) [16].

The plasmids pAAV2.1-TBG-fARSB [34] and pAAV2.1-TBG-fARSB-WPRE-m [34] were used for transfection of HepG2 cells (ATCC-LGC Standards, Milan, Italy).

AAV vectors were produced by the AAV Vector Core of the Telethon Institute of Genetics and Medicine (TIGEM, Naples, Italy) by triple transfection of 293 cells (ATCC-LGC Standards, Milan, Italy) and purified by CsCl gradients. Physical titers of the viral preparations (genome copies [gc]/ml) were determined by Real-time PCR (Applied Biosystems, Foster City, CA, USA) and dot blot analysis.

The following plasmids were used for the production of the AAV vectors used in our study:

The pAAV2.1-TBG-eGFP-WPRE-m [15] and pAAV2.1-TBG-hARSB [15] plasmids were used to produce the corresponding AAV2/8-TBG-eGFP-WPRE-m and AAV2/8-TBG-hARSB vectors. pAAV2.1-TBG-eGFP-WPRE-m was digested with BamHI and BglII (Roche, Basel, Switzerland) to remove WPRE-m and was used to produce AAV2/8-TBG-eGFP vectors.

To generate the pAAV2.1-TBG-hARSBmiR142-Tx4 plasmid, four copies of a sequence perfectly complementary to miR142-3p [35] (underlined in the primer sequence below) were cloned in the BglII restriction site between the hARSB stop codon and the polyA sequence of pAAV2.1-TBG-hARSB. The miR142-Tx4 element was constructed by annealing the following two sets of oligonucleotides:

5′-CTAGATCTTCCATAAAGTAGGAAACACTACACGATTCCATAAAGTAGGAAACACTACAAAGCTT -3′;

5′- TGTAGTGTTTCCTACTTTATGGAATCGTGTAGTGTTTCCTACTTTATGGAAGAT-3′ and

5′- TCCATAAAGTAGGAAACACTACATCACTCCATAAAGTAGGAAACACTACAAGATC -3′;

5′- TCGAGATCTTGTAGTGTTTCCTACTTTATGGAGTGATGTAGTGTTTCCTACTTTATGGAAAGCTT -3′. The resulting double-stranded fragments (each one containing two copies of miR142-T) were ligated thanks to the presence of phosphorylated 5′ ends. The obtained fragment (containing four copies of miR142-T) was subcloned in pBluescript II SK(+) previously digested with XbaI and XhoI. The recombinant clones were digested with BglII to release the fragment containing the four miR142-T sites with BglII protruding ends. To generate the pAAV2.1-TBG-eGFP-miR142-Tx4 plasmids, the eGFP coding sequence was excised from pAAV2/8-TBG-eGFP by restriction enzyme digestion with NotI and HindIII (Roche, Basel, Switzerland); the resulting eGFP fragment was then cloned in pAAV2.1-TBG-hARSBmiR142-Tx4 previously digested with NotI and ClaI to remove the hARSB coding sequence while keeping miR142-Tx4. To allow the ligation between the ClaI and HindIII non-compatible protruding ends, fill-in was performed with DNA Polymerase I, Large (Klenow) Fragment (New England Biolabs, Ipswich, MA).

### Animal procedures and vector administration

Rats from the MPS VI colony [15] mantained at Centre of Biotechnology, Animal Research Unit, Cardarelli Hospital (Naples, Italy) were used in this study. Heterozygous (het) animals were bred to produce normal (NR), het and affected (AF) off-spring. Genotype analysis was performed by polymerase chain reaction on genomic DNA as described previously [15] and the presence of the mutation was confirmed by sequencing (PRIMM, Naples, Italy).

AAV2/8 vectors were administered to NR and MPS VI rats in the temporal vein (for P4 injections) or in the femural vein (for P30 injections) at a dose of 4×10e13 gc/kg. Animals receiving AAV2/8-TBG-hARSB or AAV2/8-TBG-hARSB-miR142-Tx4 vectors received in addition 4×10e12 gc/kg of AAV2/8-TBG-eGFP-WPRE-m to confirm liver transduction. To minimize variability related to the quality of viral vector preparations, NR rats injected at P4 for the experiment in Fig. 1 received the same batch of vector. Similarly, NR and MPS VI rats injected at P30 for comparison of liver transduction efficacy (Fig. 2) received the same vector preparation. For comparison of transduction efficacy between vectors containing or not the WPRE-m, three independent groups of rats were injected with three different preparations of each vector which gave similar results (Fig. 4).

Sera from rats injected with AAV2/8-TBG-hARSB or AAV2/8-TBG-hARSB-miR142-Tx4 vectors were collected each month for analysis of ARSB activity and anti-ARSB antibodies.

At the time points reported in the Results section, animals were sacrificed and tissues were collected for subsequent analysis. For ARSB activity assays and Western blot analysis, tissues were snap frozen in dry-ice. For histological analysis, tissues were fixed in 4% PFA or 10% neutral buffered formalin (Sigma-Aldrich, Milan, Italy) and embedded in OCT compound (KALTEK, Padova, Italy). For analysis of GAG storage, tissues were fixed in methacarn (30% chloroform, 60% methanol, 10% acetic acid) and embedded in paraffin with standard procedures.

### EGFP expression and immuno-fluorescence analysis

For evaluation of eGFP expressing cells, 12 µm sections from PFA fixed, OCT-embedded livers were analyzed under a fluorescence microscope. For immuno-fluorescence with anti Albumin antibodies, 3 µm sections from PFA fixed, OCT-embedded livers were post-fixed in PFA 4% for 20′ at room temperature (RT), permeabilized with 0.3% Triton in PBS for 10′, blocked in PBS, 0.3% triton, 5% normal rabbit serum for 1 h at RT and incubated over night with sheep anti-albumin antibodies (Abcam AB98940, Cambridge, UK, 1∶100 dilution) in the same solution. After washing in PBS, the secondary antibody, diluted 1∶1000 in blocking solution, was added (594-AlexaFluor anti-sheep, Invitrogen Corporation, Carlsbad, CA, USA).

Staining with anti-CD163 antibodies was similarly performed on 3 µm PFA fixed liver sections blocked O.N. with PBS, 0.1% triton, 10% FBS, 0.1% BSA, incubated for 2 h at RT with mouse anti-CD163 antibodies (1∶100 dilution in the same solution, Serotech MCA342r, Oxford, UK) and then with the secondary antibody (diluted 1∶1000, 594-AlexaFluor, anti-mouse, Invitrogen Corporation, Carlsbad, CA, USA). Immuno-fluorescence with mouse anti-CD31 antibodies (Serotech MCA1334, Oxford, UK) was performed on 5 µm frozen sections from OCT-embedded formalin-fixed livers. Sections were blocked O.N. with PBS, 0.1% triton, 5% FBS, 1% BSA, incubated for 2 h at RT with mouse anti-CD31 antibodies (diluted 1∶100 in PBS, 0.1% triton, 1% FBS, 1% BSA) and then with the secondary antibody (diluted 1∶1000 in the same solution, 594-AlexaFluor, anti-mouse, Invitrogen Corporation, Carlsbad, CA, USA). Slides were mounted with vectashield medium (Vectorlabs, Burlingame, CA, USA) and pictures were taken with a confocal microscope (LSM510, Carl Zeiss, Milan, Italy).

### Western blot analysis

For Western blot analysis, tissues were homogenized in RIPA buffer (50 mM NaCL, 25 mM TrisHCl pH 8.0, 0.5% NP40, 0.1% SDS, 10 mg/ml leupeptin–aprotinin– pepstatin A) with a tissue lyser (Qiagen). Protein concentration was determined with the BCA protein assay reagent (Pierce Chemical Co, Boston, MA, USA); 50 µg of lysates for the liver, 70 µg for kidney and spleen and 150 µg for gonads, heart and gastrocnemious were separated by SDS page on a 14% polyacrylamide gel, transferred to a PVDF membrane (Roche, Basel, Switzerland) and incubated with anti eGFP antibodies (Santa Cruz Biotechnology, Santa Cruz, CA, 1∶500 dilution). An HRP-labeled anti rabbit antibody (1∶1000 dilution) was then used for detection (Amersham Biosciences, Piscataway, NJ, USA). Filters were then stripped and reprobed with anti-Tubulin antibodies (Sigma-Aldrich, Milan, Italy, 1∶1000 dilution).

Western blot band intensity was quantified with the ImageJ 1.38× software (http://rsb.info.nih.gov) and the intensity of eGFP bands was normalized on the corresponding tubulin band and used for comparisons.

### AAV vector genome distribution

Genomic DNA was extracted from snap frozen tissues after lysis in a buffer containing 10 mM Tris, 10 mM EDTA, 0.6% SDS, 200 ng/µl proteinase K. RNAseA (Qiagen, Washington, DC, USA) was added at a final concentration of 10 µg/µl and samples were incubated at 37°C for 1 h followed by two rounds of phenol/chloroform extractions. DNA was then precipitated by adding 2.5 volumes of EtOH and incubated at −80°C for 2 h. Samples were centrifuged at 14000 rpm for 1 h, DNA pellets were washed in 70% EtOH and resuspended in water.

Real-time PCR analysis was performed on 100 ng of genomic DNA using a set of primers/probe specific for the viral genome and the Taqman universal PCR master mix (Applied Biosystems, Foster City, CA, USA) [34]. Amplification was run on a 7900 Real-time PCR system (Applied Biosystems, Foster City, CA, USA) with standard cycles. All the reactions were performed in triplicate. Liver, spleen, kidney, muscle, heart and gonads were colleted at P90 for rats injected at P30 and at P15, P30 and P90 for rats injected at P4.

### ELISA for the detection of anti-ARSB antibodies

The presence of circulating antibodies against the hARSB protein in MPS VI rats was determined using a plate-binding assay, as described previously [15]. Briefly, 96-well 200 microtiter plates (Costar, Cambridge, UK) were coated with human ARSB (Naglazyme - Galsulfase, BioMarin Europe Ltd., London, UK) in 100 µl of PBS overnight at 4°C. Plates were blocked for 2 h at room temperature (RT) with 200 µl 100% FBS, washed five times with PBS containing 0.05% Tween 20 and incubated with 100 ul of 1∶50 dilutions of rat serum in PBS/Tween 0.05%/FBS 10% for 2 h at RT. The washing step was repeated once and 100 µl of biotinylated anti-rat IgG antibody (Vector Laboratories Ltd., Peterborough, UK) was added in PBS/Tween 0.05%/FBS 10% for 1 h at RT. Binding was revealed by the addition of extravidin–horseradish peroxidase (Sigma-Aldrich, Milan, Italy) followed by o-phenylenediamine dihydrochloride (OPD) substrate (Sigma-Aldrich, Milan, Italy). Absorbance at 450 nm (OD) was determined in an Infinite 200 microplate reader (Tecan, Mannedorf, Switzerland). The OD observed in an assay represents a qualitative measure of antibody affinity. As control we used sera from normal and affected rats who did not receive the vector (n = 18); the average OD measured in control sera was considered as the cut-off value for detection of anti-ARSB antibody presence.

### Alcian-blue staining

7 µm paraffin sections from methacarn fixed livers were rehydrated and stained with 1% Alcian blue (Sigma-Aldrich, Milan, Italy) in hydrochloric acid. The counterstaining was performed for 2 min with Nuclear-Fast red (Sigma-Aldrich, Milan, Italy) and slides were mounted with Eukitt (Kaltek, Padua, Italy).

### ARSB activity assay and protein levels

Livers from injected and control rats were lysed in water by freeze and thaw and protein concentration was determined using the BCA protein assay reagent (Pierce Chemical Co, Boston, MA, USA).

The ARSB assay was performed as described previously [15]. Briefly, 15–30 µg of protein or 20 µl of serum (diluted 1∶2 with water) were incubated with 40 µl of fluorogenic substrate, 4-methylumbelliferyl-sulfate (12.5 mM Sigma-Aldrich, Milan, Italy), for 3 h at 37°C in presence of 40 µl silver nitrate (0.75 mM, Carlo Erba, Milan, Italy), both dissolved in NaOAc, 0.2 M, pH 5.6. The reaction was stopped by adding 200 µl of the carbonate stop buffer (0.5 M NaHCO3/0.5 M Na2CO3, pH 10.7), and the fluorescence of the 4-methylumbelliferone liberated was measured in an Infinite 200 microplate reader (Tecan, Mannedorf, Switzerland) using 365 nm excitation and 460 nm emission. The enzyme activities were calculated using a standard curve of the fluorogenic product, 4-methylumbelliferone (Sigma-Aldrich, Milan, Italy). For tissue lysates and cells the activity is expressed as nmol/mg protein/h. For serum samples, enzyme activity is calculated as % of activity measured in serum from NR rats in each assay (reported as 100%).

### Cell culture conditions, transfection, AAV infection and fluorescence detection

Primary human umbilical vein endothelial cells (HUVECs; Promocell, Heidelberg, Germany) were cultivated according to the manufacturer's instructions.

HepG2 cells (ATCC-LGC Standards, Milan, Italy) were grown in Dulbecco's modified Eagle medium (DMEM, Celbio, Milan, Italy) supplemented with 10% (w/v) FBS (GIBCO; Invitrogen Corporation, Carlsbad, CA, USA), 4.5 g/l glucose, 2 mM glutamine and penicillin (100 IU/ml)-streptomycin (10 µg/ml) (Invitrogen Corporation, Carlsbad, CA, USA). All cells were cultivated in a humidified incubator at 5% CO2 at 37°C.

The day before transfection HepG2 cells were plated in 6 well plates (MW6) and transfected the day after with 2 µg of pAAV2.1-TBG-eGFP or pAAV2.1-TBG-eGFP-WPRE-m using the Fugene reagent (Roche, Basel, Switzerland). Fourty-eight hours after transfection fluorescence intensity (OD) was measured in each well with an Infinite 200 microplate reader (Tecan, Mannedorf, Switzerland) using 485 nm excitation and 535 nm emission.

Pictures of each well containing eGFP-positive cells were taken with a fluorescence microscope. All transfections were performed in duplicate.

For transfection with ARSB expressing plasmids, HepG2 cells were plated in MW6 and transfected with 1.5 ug of pAAV2.1-TBG-felineARSB plasmids containing or not WPRE-m using Fugene reagent (Roche). To normalize transfection efficacy, cells in each well received 0.5 ug of pAAV2.1-CMV-eGFP plasmids. 48 h after transfection cells were incubated in serum free medium; 24 hours later eGFP fluorescence in each well was measured with an Infinite 200 microplate reader (Tecan, Mannedorf, Switzerland); cells and media were then collected for analysis of ARSB activity which was normalized on eGFP fluorescence intensity for each sample by dividing the ARSB activity measured (nmol/mg/h) for the eGFP fluorescence intensity (OD).

For infection with AAV vectors, HepG2 and HUVEC cells were seeded 24 hrs prior to transduction. Cells were incubated with the indicated vector preparation at a genomic particle per cell ratio of 1×10e5 and 5×10e5, respectively. Cells were harvested 48 hrs post transduction by trypsin treatment. Transgene-expressing cells were determined by flow cytometry using a BD FACSCanto II (BD Biosciences, San Jose, CA). A minimum of 10000 cells was counted for each sample. The background fluorescence level was set to 1%.

### RNA extraction and RT-PCR for detection of eGFP transcript

Liver, spleen and kidney of rats injected at P4 with AAV2/8-TBG-eGFP vectors were collected at P15; total RNA was isolated with TRIzol reagent (Invitrogen, Carlsbad, CA) as suggested by the manufacturer and subjected to DNAse I (Invitrogen, Carlsbad, CA) treatment to eliminate contaminant genomic DNA. cDNA was synthesized from 1 µg of RNA using SuperScript III First-Strand Synthesis System (Invitrogen, Carlsbad, CA) and then subjected to RNAseH digestion (Invitrogen, Carlsbad, CA). RT-PCR was performed on 1 µl of each cDNA with the following primers: eGFP-Fw: 5′-GACGGCAACTACAAGACC-3′; eGFP-Rev: 5′-CTTGATGCCGTTCTTCTGC-3′; Actin-Fw: 5′-GACCTCTATGCCAACACAG-3′; Actin-Rev: 5′ GAGCCACCAATCCACACAG-3′.

### Statistical analyses

An unpaired *t-*test between the various data sets was performed using the Microsoft Excel (Microsoft Corporation, Redmond, VA, USA) *t*-test function. Significance at *p*≤0.05 is indicated by a single asterisk in the figures.

## Supporting Information

Figure S1 **Analysis of eGFP expression in livers of rats injected with AAV2/8-TBG-eGFP and collected at P90.** A) Wild-type rats were injected at either postnatal day 4 (P4, upper pictures), or at P30 (lower pictures) with 4×10e13 gc/kg of AAV2/8-TBG-eGFP. Animals were sacrificed at P90 and eGFP expression was confirmed under a fluorescence microscope on sections from transduced livers. Pictures from representative animals in each group are shown. Magnification: 20×. B) The eGFP band intensity from Western blot analysis of livers from animals in panel A and shown in Fig. 1B was reported on a linear scale. The intensity of eGFP bands was quantified, normalized on the corresponding tubulin band and expressed as fold of increase compared to rats injected at P4 and sacrificed at P90. Results are reported as mean ± SE. The number of rats analyzed in each group is reported in each bar.
(TIF)

Figure S2 **Western Blot analysis of eGFP expression in livers of rats injected with AAV2/8-TBG-eGFP.** A, B, C) Wild-type rats were injected at either postnatal day 4 (P4) or at P30 with 4×10e13 gc/kg of AAV2/8-TBG-eGFP. Animals injected at P4 were sacrificed at P15, P30 or P90 (A and B), while those injected at P30 were analysed both at P41 (C) and at P90 (A and C). EGFP expression was compared among rats of the different groups by Western blot analysis with anti-eGFP antibodies. Protein loading was normalized by blotting with anti Tubulin (α-Tub) antibodies. Time points of vector administration and liver collection are reported in each picture. Representative Western blots are shown for each animal group. D) Wild-type (NR) and MPS VI (AF) rats were injected at P30 with 4×10e13 gc/kg of AAV2/8-TBG-eGFP vectors. Animals were sacrificed at P90 and eGFP expression was analyzed by Western blot analysis with anti-eGFP antibodies. Protein loading was normalized by blotting with anti Tubulin (α-Tub) antibodies. E) Wild-type rats were injected at postnatal day (P)30 with 4×10e13 gc/kg of AAV2/8-TBG-eGFP or AAV2/8-TBG-eGFP-WPRE-m vectors. Animals were sacrificed at P90 and eGFP expression was analyzed by Western blot analysis with anti-eGFP antibodies. Protein loading was normalized by blotting with anti Tubulin (α-Tub) antibodies. Representative Western blots are shown from three independent experiments. F) Wild-type rats were injected at postnatal day (P)4 with 4×10e13 gc/kg of either AAV2/8-TBG-eGFP or AAV2/8-TBG-eGFP-miR142-Tx4 vectors. Animals were sacrificed at P15 and eGFP expression in liver and spleen was analyzed by Western blot with anti-eGFP antibodies. Protein loading was normalized by blotting with anti Tubulin (α-Tub) antibodies.
(TIF)

Figure S3 **FACS analysis of eGFP-expressing human hepatoma and endothelial cells after transduction with AAV2/8.** Human hepatoma (HepG2) and human umbilical vein endothelial (HUVEC) cells were infected with 5×10e5 gc/cell of AAV2/8-TBG-eGFP (black bars) or AAV2/8-CBA-eGFP (white bars) vectors. The percentage of eGFP expressing cells was determined by FACS analysis. Results are reported as mean ± SE of three experiments.
(TIF)

Figure S4 **RT-PCR analysis of eGFP expression in liver, spleen and kidney of rats injected with AAV2/8-TBG-eGFP.** Wild-type rats were injected at postnatal day (P) 4 with 4×10e13 gc/kg of AAV2/8-TBG-eGFP. Tissues were collected at P15, RNA was isolated, retrotranscribed (RT+) and PCR-amplified with eGFP- or Actin-specific primers. As control, non-retrotranscribed RNA (RT−) was amplified for each sample in the same conditions. Tissues from a non-injected rat (−) were used as negative control.
(TIF)

Figure S5 **Scatter plots of eGFP expression levels and AAV vector genome copies from liver of rats injected with AAV2/8-TBG-eGFP containing or not WPRE-m.** The eGFP Western blot band intensity normalyzed on tubulin (eGFP/Tubulin, A) and AAV vector genome copies/molecule of diploid genome (gc/mdg, B) from liver of animals shown in Fig. 4 were represented as Scatter Plot. Mean ± SE for each experimental group is shown. *: p<0.05.
(TIF)
