# Supplementary figures and images for: Impact of Age at Administration, Lysosomal Storage, and Transgene Regulatory Elements on AAV2/8-Mediated Rat Liver Transduction
Source: PLoS One. 2012 Mar 13;7(3):e33286. doi: 10.1371/journal.pone.0033286 (PMC3302848; doi:10.1371/journal.pone.0033286)

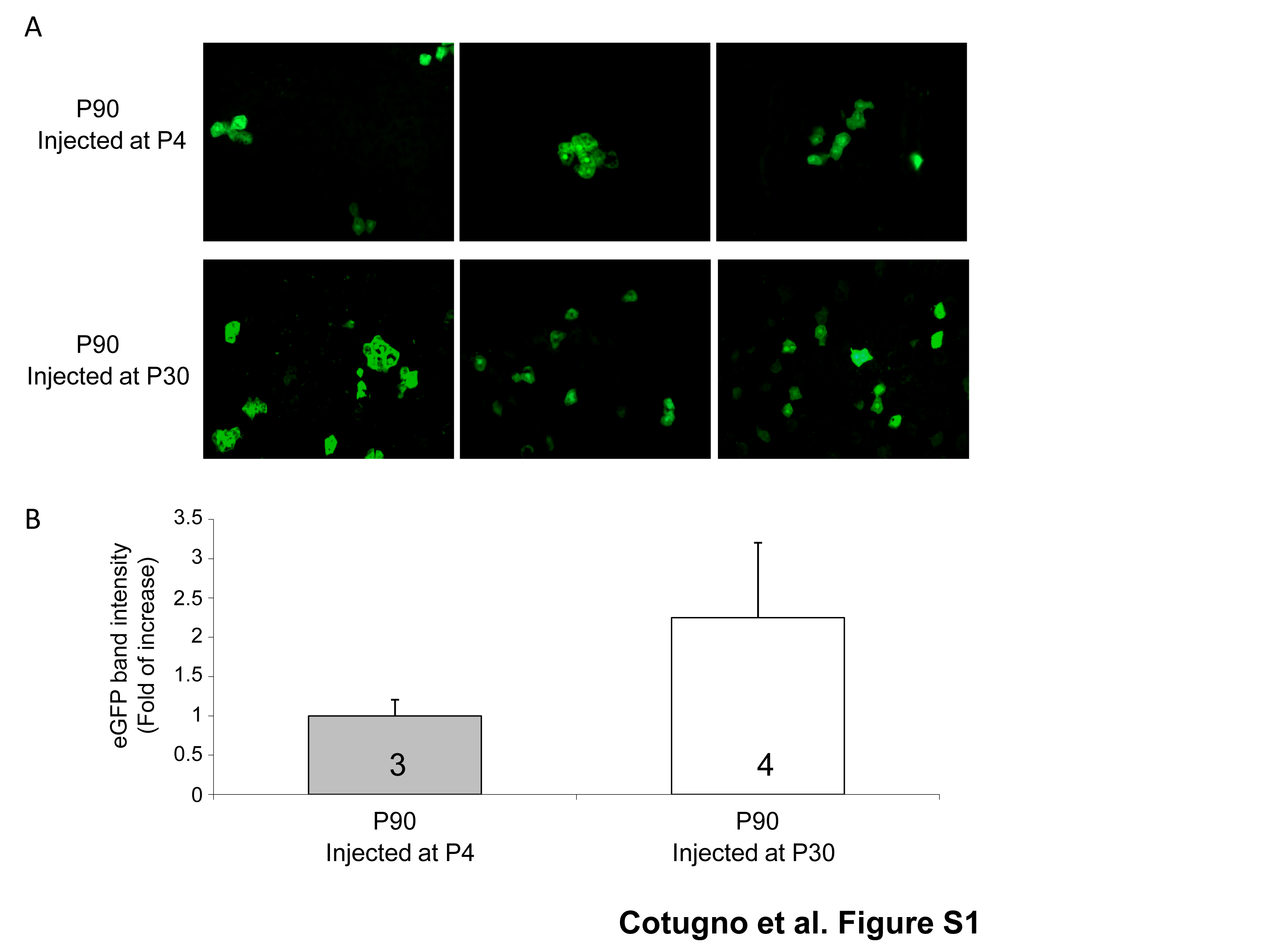

Supplement: Figure S1 — Analysis of eGFP expression in livers of rats injected with AAV2/8-TBG-eGFP and collected at P90. A) Wild-type rats were injected at either postnatal day 4 (P4, upper pictures), or at P30 (lower pictures) with 4×10e13 gc/kg of AAV2/8-TBG-eGFP. Animals were sacrificed at P90 and eGFP expression was confirmed under a fluorescence microscope on sections from transduced livers. Pictures from representative animals in each group are shown. Magnification: 20×. B) The eGFP band intensity from Western blot analysis of livers from animals in panel A and shown in Fig. 1B was reported on a linear scale. The intensity of eGFP bands was quantified, normalized on the corresponding tubulin band and expressed as fold of increase compared to rats injected at P4 and sacrificed at P90. Results are reported as mean ± SE. The number of rats analyzed in each group is reported in each bar. (TIF) [file pone.0033286.s001.tif]

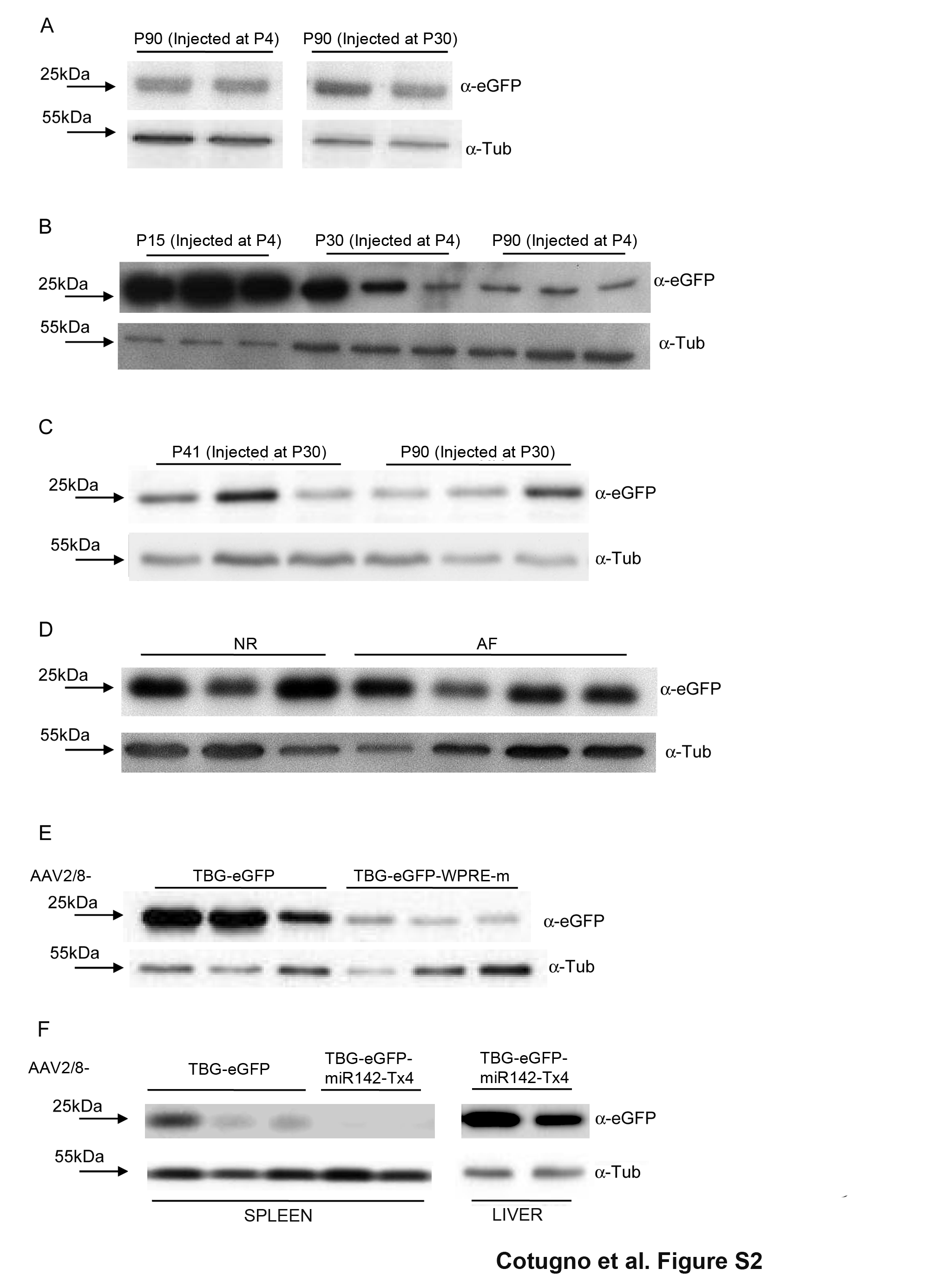

Supplement: Figure S2 — Western Blot analysis of eGFP expression in livers of rats injected with AAV2/8-TBG-eGFP. A, B, C) Wild-type rats were injected at either postnatal day 4 (P4) or at P30 with 4×10e13 gc/kg of AAV2/8-TBG-eGFP. Animals injected at P4 were sacrificed at P15, P30 or P90 (A and B), while those injected at P30 were analysed both at P41 (C) and at P90 (A and C). EGFP expression was compared among rats of the different groups by Western blot analysis with anti-eGFP antibodies. Protein loading was normalized by blotting with anti Tubulin (α-Tub) antibodies. Time points of vector administration and liver collection are reported in each picture. Representative Western blots are shown for each animal group. D) Wild-type (NR) and MPS VI (AF) rats were injected at P30 with 4×10e13 gc/kg of AAV2/8-TBG-eGFP vectors. Animals were sacrificed at P90 and eGFP expression was analyzed by Western blot analysis with anti-eGFP antibodies. Protein loading was normalized by blotting with anti Tubulin (α-Tub) antibodies. E) Wild-type rats were injected at postnatal day (P)30 with 4×10e13 gc/kg of AAV2/8-TBG-eGFP or AAV2/8-TBG-eGFP-WPRE-m vectors. Animals were sacrificed at P90 and eGFP expression was analyzed by Western blot analysis with anti-eGFP antibodies. Protein loading was normalized by blotting with anti Tubulin (α-Tub) antibodies. Representative Western blots are shown from three independent experiments. F) Wild-type rats were injected at postnatal day (P)4 with 4×10e13 gc/kg of either AAV2/8-TBG-eGFP or AAV2/8-TBG-eGFP-miR142-Tx4 vectors. Animals were sacrificed at P15 and eGFP expression in liver and spleen was analyzed by Western blot with anti-eGFP antibodies. Protein loading was normalized by blotting with anti Tubulin (α-Tub) antibodies. (TIF) [file pone.0033286.s002.tif]

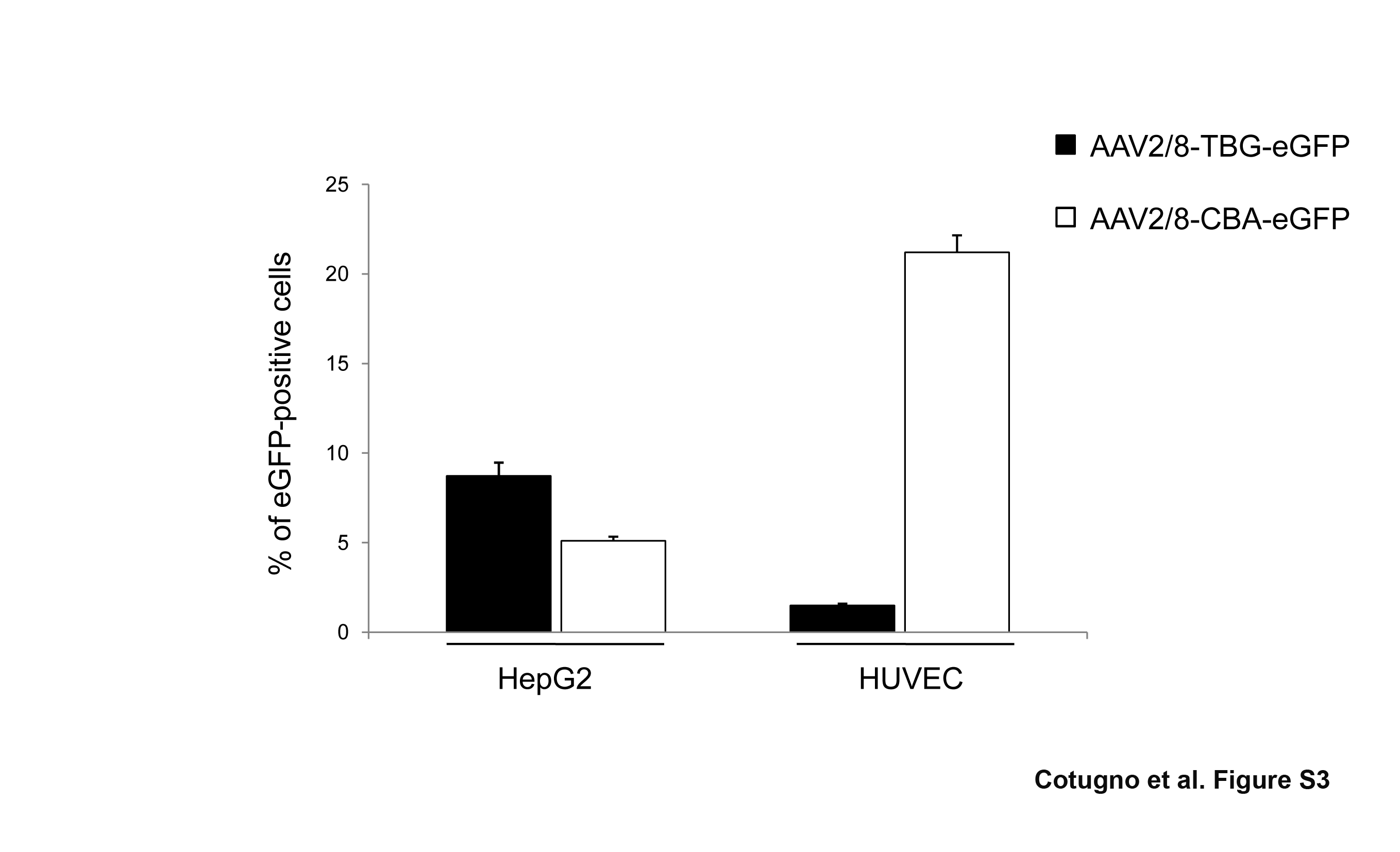

Supplement: Figure S3 — FACS analysis of eGFP-expressing human hepatoma and endothelial cells after transduction with AAV2/8. Human hepatoma (HepG2) and human umbilical vein endothelial (HUVEC) cells were infected with 5×10e5 gc/cell of AAV2/8-TBG-eGFP (black bars) or AAV2/8-CBA-eGFP (white bars) vectors. The percentage of eGFP expressing cells was determined by FACS analysis. Results are reported as mean ± SE of three experiments. (TIF) [file pone.0033286.s003.tif]

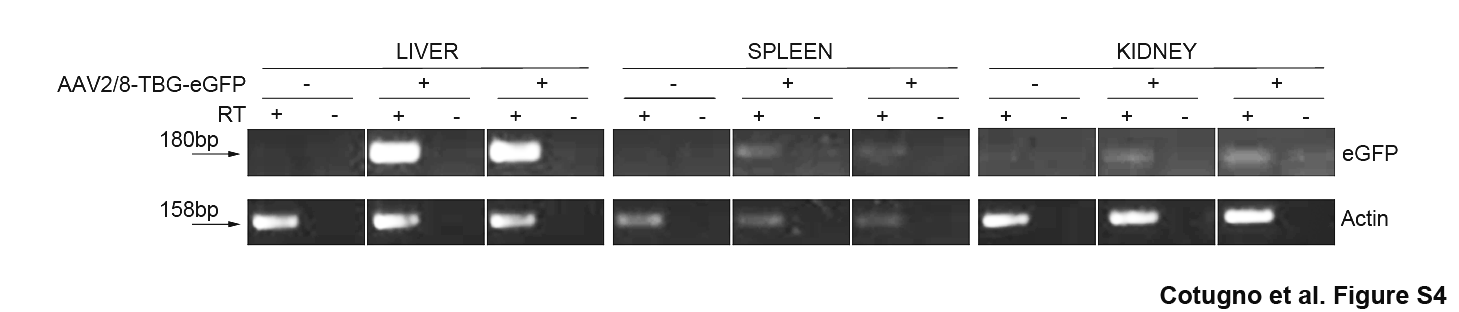

Supplement: Figure S4 — RT-PCR analysis of eGFP expression in liver, spleen and kidney of rats injected with AAV2/8-TBG-eGFP. Wild-type rats were injected at postnatal day (P) 4 with 4×10e13 gc/kg of AAV2/8-TBG-eGFP. Tissues were collected at P15, RNA was isolated, retrotranscribed (RT+) and PCR-amplified with eGFP- or Actin-specific primers. As control, non-retrotranscribed RNA (RT−) was amplified for each sample in the same conditions. Tissues from a non-injected rat (−) were used as negative control. (TIF) [file pone.0033286.s004.tif]

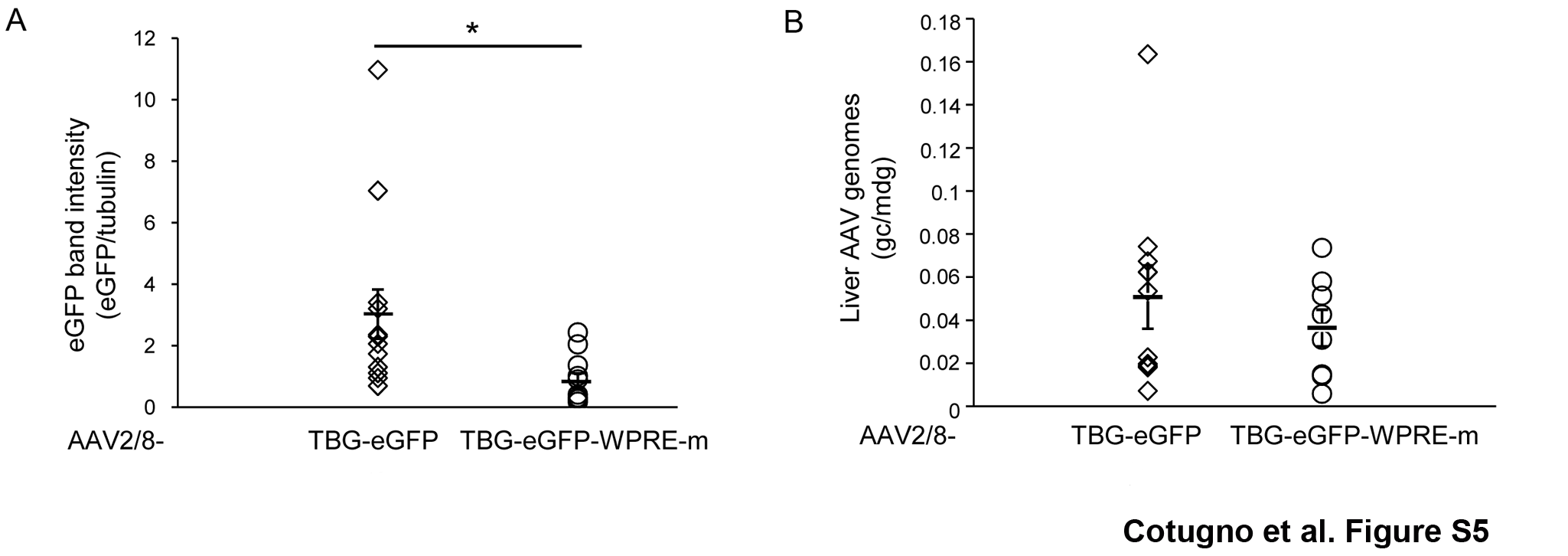

Supplement: Figure S5 — Scatter plots of eGFP expression levels and AAV vector genome copies from liver of rats injected with AAV2/8-TBG-eGFP containing or not WPRE-m. The eGFP Western blot band intensity normalyzed on tubulin (eGFP/Tubulin, A) and AAV vector genome copies/molecule of diploid genome (gc/mdg, B) from liver of animals shown in Fig. 4 were represented as Scatter Plot. Mean ± SE for each experimental group is shown. *: p<0.05. (TIF) [file pone.0033286.s005.tif]
